# Influence of the Active Site Flexibility on the Efficiency of Substrate Activation in the Active Sites of Bi-Zinc Metallo-β-Lactamases

**DOI:** 10.3390/molecules27207031

**Published:** 2022-10-18

**Authors:** Alexandra V. Krivitskaya, Maria G. Khrenova

**Affiliations:** 1 Bach Institute of Biochemistry, Federal Research Centre “Fundamentals of Biotechnology” of the Russian Academy of Sciences, 119071 Moscow, Russia; 2 Department of Chemistry, Interdisciplinary Scientific and Educational School of Moscow University «Brain, Cognitive Systems, Artificial Intelligence», Lomonosov Moscow State University, 119991 Moscow, Russia

**Keywords:** metallo-β-lactamase, QM/MM molecular dynamics, imipenem, active site flexibility, substrate activation, machine learning

## Abstract

The influence of the active site flexibility on the efficiency of catalytic reaction is studied by taking two members of metallo-β-lactamases, L1 and NDM-1, with the same substrate, imipenem. Active sites of these proteins are covered by L10 loops, and differences in their amino acid compositions affect their rigidity. A more flexible loop in the NDM-1 brings additional flexibility to the active site in the ES complex. This is pronounced in wider distributions of key interatomic distances, such as the distance of the nucleophilic attack, coordination bond lengths, and covalent bond lengths in the substrate. Substrate activation, quantified by Fukui electrophilicity index of the carbonyl carbon atom of the substrate, is also sensitive to the active site flexibility. In the tighter and more rigid L1 enzyme-substrate complex, the substrate is activated more efficiently. In the NDM-1 containing system, only one third of the states are activated to the same extent. Other fractions demonstrate lower substrate activation. Efficiency of the substrate activation and rigidity of the ES complex influence the following chemical reaction. In the more rigid L1-containing system, the reaction barrier of the first step of the reaction is lower, and the first intermediate is more stabilized compared to the NDM-1 containing system.

## 1. Introduction

Enzymes that belong to the same family share similar composition of active sites. Still, differences in the overall structure may affect their flexibility and particular conformations of the active site residues. The simplest model of the enzyme-substrate interaction is the “lock and key”, which assumes that the enzyme is quite rigid and compatible with a certain substrate. The idea of the enzyme flexibility was introduced later by Koshland in his “induced fit” model [1]. Accumulation of more than 60,000 crystal structures of different enzymes in the protein data bank makes it possible to perform big data analysis and explicitly demonstrate flexibility of amino acid residues [2,3,4,5,6]. These studies are based on the analysis of crystal structures and are related to the stable states like apo and holo forms, enzyme-inhibitor complexes, etc., but not metastable reactive species.

**Figure 1:**
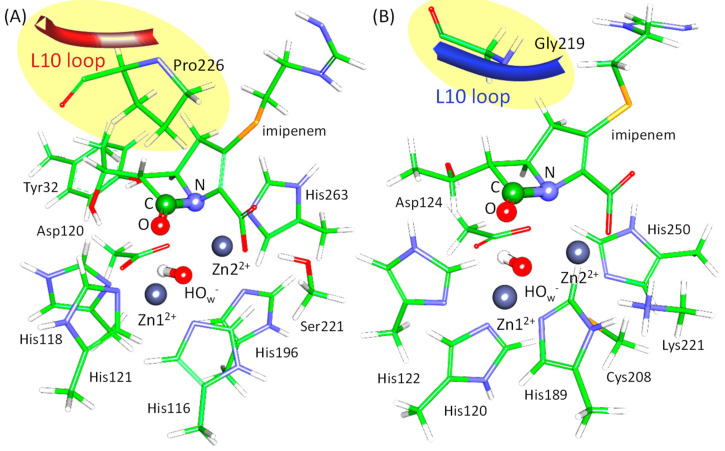
Active sites of the L1 (**A**) and the NDM-1 (**B**) with imipenem. The fragment of the L10 loop is shown in red for the L1 and in dark blue for the NDM-1. Coordination spheres of zinc cations and side chains of amino acid residues forming hydrogen bonds with the carboxyl group of imipenem are depicted with thin sticks. The N–C=O fragment of the β-lactam ring that participates in chemical reaction is shown in thick sticks. Here, carbon is green, oxygen is red, nitrogen is blue, hydrogen is white, sulfur is yellow, and zinc is dark gray.

These studies encouraged us to deepen the understanding of the role of active site flexibility, focusing on the reactive states. To do this, we selected two bacterial enzymes related to the antibiotic resistance, metallo-β-lactamases L1 (a B3 metallo-β-lactamase from *Stenotrophomonas* (*Xanthomonas*) *maltophilia*) and NDM-1 (New Delhi metallo-β-lactamase), belonging to the same B class [7]. The reaction mechanism of the imipenem antibiotic hydrolysis by these two enzymes has already been studied [8]. The consequence of the elementary steps is the same for both enzymes; still, the energy barrier at the first step is lower for the L1-containing system. Also, the first intermediate is stabilized relative to the enzyme-substrate complex (ES) in the L1-imipenem complex and destabilized in the NDM-1. In both enzymes, the L10 loop that is conservative for metallo-β-lactamases covers the substrate and fixes it in the active site (Figure 1, [9]). In the L1, this loop contains a proline residue right above the substrate that brings rigidity to the construct (Figure 1A). Conversely, in the NDM-1, this loop is more flexible, as it carries glycine residue at the same position, and the pyrroline ring of imipenem is more exposed to the solution in the NDM-1 (Figure 1B). Both enzymes contain two zinc cations, Zn1^2+^ and Zn2^2+^, bridged by a catalytic hydroxide ion, O_w_H^−^ (Figure 1). Ligands of Zn1^2+^ cations are the same in both enzymes: three histidine residues, catalytic hydroxide anion, and a carbonyl group of the substrate. The coordination surrounding the Zn2^2+^ cation differs only in one ligand: a histidine residue in the L1 and a cysteine residue in the NDM-1. Others are the same; those are a histidine residue, a catalytic O_w_H^−^, a catalytic aspartate residue, and a nitrogen atom of the substrate (Figure 1).

**Figure 2:**
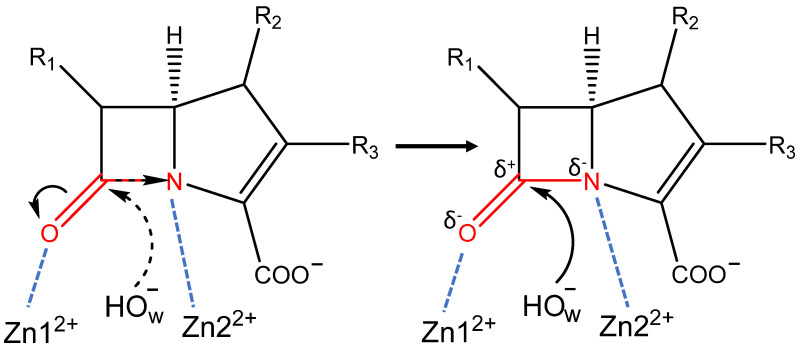
Scheme of the activation of the carbapenem by metallo-β-lactamases L1 and NDM-1. Black arrows on the left part of the figure shows the direction of charge concentration shift; on the right side, the nucleophilic attack of the activated carbonyl carbon by O_w_H^−^ is shown. Black dashed arrow shows the nucleophilic attack on the non-activated carbonyl carbon, blue dashed lines show coordination bonds of zinc ions with the substrate in the active site. Red molecular fragment polarizes in the active site, thus activating the carbonyl carbon atom.

In enzyme-substrate complexes of both enzymes, the oxyanion hole is formed by the Zn1^2+^ cation, and its interactions with the carbonyl oxygen atom polarize the C=O bond of the substrate (Figure 2). Additionally, the neighboring C-N bond is polarized due to the interactions between its nitrogen atom and a Zn2^2+^ cation (Figure 2). These interactions prepare or activate the carbonyl carbon atom of the β-lactam ring for nucleophilic attack that initiates the reaction. Polarization of both C=O and C-N bonds results in the increase of the negative partial charges on heteroatoms (δ^−^) and increase of the partial positive charge (δ^+^) on the carbon atom. Thus, the carbonyl carbon becomes more electrophilic, which facilitates nucleophilic attack by the catalytic O_w_H^−^. The nucleophilic particle O_w_H^−^ attacks a carbonyl carbon atom, leading to the formation of the tetrahedral intermediate. Substrate activation can be quantified in different ways, including simple geometry criteria and more complicated electron density-based descriptors. Interatomic distances in the oxyanion hole and distances of the nucleophilic attack can be utilized as a measure of interactions of the carbonyl group of the substrate with the active site of the enzyme. Among electron density-based criteria, here, we focus on Laplacian of electron density [10,11,12,13,14] and Fukui atomic indices [15,16,17] (for their applications see, for example, refs [13,18,19,20,21,22]). Analysis of Laplacian of electron density, ∇^2^ρ(r), allows identification of the areas of the local electronic charge concentration with ∇^2^ρ(r) < 0 (nucleophilic sites) and electronic charge depletion areas with ∇^2^ρ(r) > 0 (electrophilic sites) in molecular systems. Analysis of ∇^2^ρ(r) 2D maps in the plane formed by the nucleophilic atom of the catalytic particle and the carbonyl group of the substrate provides easily visible images with a binary classification as reactive and nonreactive species. Fukui atomic indices are utilized for quantitative estimates of electrophilic and nucleophilic site. Here we use Fukui electrophilicity index to quantify the extent of activation of the carbonyl carbon atom of the substrate.

In this study, we compare the flexibility of two enzyme-substrate complexes and reveal how the flexibility affect the energetic of the subsequent chemical reaction. We rely on both geometry criteria and electron density-based features. We calculate interatomic distances in the enzymatic active site and obtain electron density-based features that are responsible for reactivity in sets of QM/MM MD frames. We utilize Fukui electrophilicity index and Laplacian of electron density as descriptors and analyze how geometry criteria are interrelated with the substrate activation quantified by the Fukui electrophilicity index.

## 2. Results and Discussion

### 2.1. Dynamic Behavior of the ES Complexes: Geometry Parameters

Dynamic behavior of the ES complexes can shed light on the subsequent chemical reaction. The most straightforward approach is to analyze key interatomic distances and compare them among similar systems. Here, we compare dynamic behavior of the ES complexes of the L1 (L1-ES) and NDM-1 (NDM-1-ES) metallo-β-lactamases with the imipenem substrate. 

**Figure 3:**
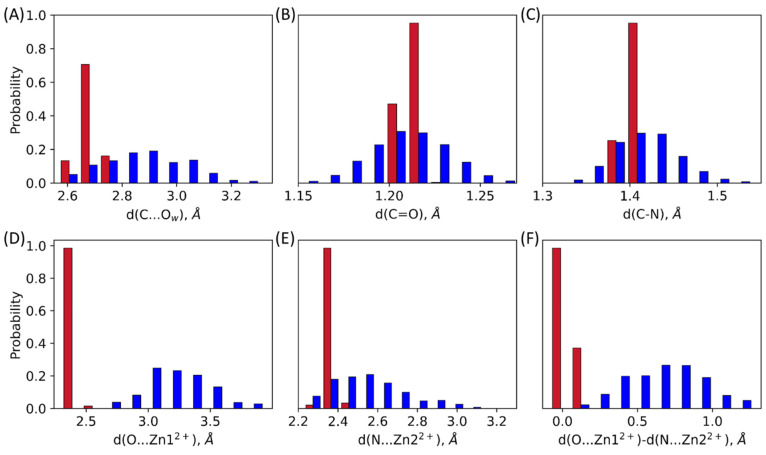
Distributions of interatomic distances in the L1-ES (red) and the NDM-1-ES (blue): (**A**) distance of the nucleophilic attack, d(C…O_w_); (**B**) C=O bond length in the carbonyl fragment of the β-lactam ring, d(C=O); (**C**) C-N bond length of the β-lactam ring, d(C-N); (**D**) coordination bond length between the zinc cation and the oxygen atom of the carbonyl group of the substrate, d(Zn1^2+^…O) (**E**) coordination bond length between the zinc cation and the nitrogen atom of the β-lactam ring, d(Zn2^2+^…N) (**F**) difference between Zn1^2+^…O and Zn2^2+^…N coordination bond lengths. All values are from QM/MM MD trajectories of the ES complexes.

Interactions between the oxyanion hole and the carbonyl oxygen atom play a decisive role in the preparation of the substrate for a nucleophilic attack [23,24,25]. In both ES complexes, the oxyanion hole is represented by a Zn1^2+^ cation that forms a coordination bond with the carbonyl oxygen atom of the substrate (Figure 2 and Figure 3D, Table 1). Also, there is another interaction that should increase electrophilicity of the carbonyl carbon atom due to the polarization of the C-N bond of the substrate: a coordination bond between Zn2^2+^ and the nitrogen atom of the β-lactam ring (Figure 2 and Figure 3E, Table 1). In the L1-ES, d(Zn1^2+^…O) and d(Zn2^2+^…N), distributions are narrow, the distances vary within 0.21 Å and 0.14 Å, respectively. The average values are similar, being 2.39 ± 0.04 Å for the Zn1^2+^…O distance and 2.36 ± 0.02 Å for the Zn2^2+^…N distance. These values correspond to coordination bonds. In the NDM-1-ES, the distributions are much wider, the bond lengths vary within 1.32 Å and 0.9 Å for d(Zn1^2+^…O) and d(Zn2^2+^…N), respectively. Moreover, the Zn1^2+^…O distances reach the values of 4 Å. Practically, no coordination bond exists between the zinc cation and the oxygen atom of the substrate, and the only influence is due to the large positive charge of the metal cation, that is, the C=O bond polarization occurs due to the electrostatic field of the Zn1^2+^. The Zn2^2+^…N distance is shorter than Zn1^2+^…O distance; their mean values are 2.52 ± 0.18 Å and 3.18 ± 0.26 Å, respectively. We assume that the Zn2^2+^…N coordination bond may partially compensate the absence of the Zn1^2+^…O coordination bond. The C-N bond polarization should also increase the extent of the charge deconcentration on the carbonyl carbon atom in the NDM-1, thus making it an electrophile prepared for the reaction. 

The distance of the nucleophilic attack between the carbonyl carbon atom of the substrate and the catalytic O_w_H^−^ is another important feature of the ES complex. In the L1-ES, the average value of the nucleophilic attack distance is 2.67 ± 0.03 Å, the distribution is narrow, values vary in a range of 2.62–2.76 Å. For the NDM-1, the distribution is much wider, the range is 2.57–3.30 Å, the average and the standard deviation are 2.85 ± 0.15 Å. Mean values for both enzymes are close, but the shape of the distributions is considerably different (Figure 3A). In the L1-ES, distance of the nucleophilic attack varies within only 0.14 Å; for the NDM-1-ES, it increases to 0.73 Å. 

**Figure 4:**
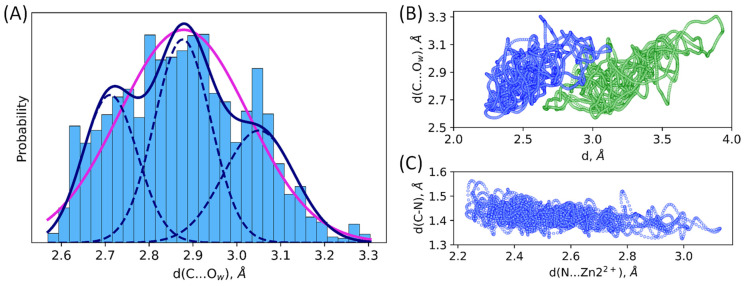
(**A**) One-state and three-state models of distribution of the nucleophilic attack distance, C…O_w_, in the NDM-1-ES. Magenta line corresponds to the unimodal normal distribution, and navy solid line is a sum of three normal distributions (navy dashed lines) with the corresponding weights. Scatter plots of interatomic distances in the MD trajectory of the NDM-1-ES; (**B**) distance of the nucleophilic attack, d(C…O_w_) vs. Zn1^2+^…O (green) or Zn2^2+^…N (blue) coordination bond, d; (**C**) C–N bond vs. Zn2^2+^…N bond.

Covalent bonds that are polarized in the active sites are also considered. Those are C=O bond and C-N bond of the β-lactam ring. Similarly to the distances distributions corresponding to the noncovalent interaction, we observe narrow distributions for the L1-ES and wide distributions for the NDM-1-ES for both C=O and C-N bonds (Figure 3B,C, Table 1). Despite the different shapes of the carbonyl bond length distributions, average values are the same for both enzymes. Those are 1.212 ± 0.003 Å and 1.210 ± 0.021 Å for the L1-ES and the NDM-1-ES, respectively. The maximum of the C-N bond length distribution is shifted towards larger values in the NDM-1-ES compared with the L1-ES. The average values here are 1.402 ± 0.007 Å and 1.418 ± 0.036 Å for the L1-ES and the NDM-1-ES, respectively. The difference of 0.16 Å in the average values is significant for a covalent bond in a cyclic fragment of a molecule. The origin of this difference might lie in the cooperative effect of coordination bonds with zinc cations (Figure 3F). To demonstrate this, we calculate the difference between d(Zn1^2+^…O) and d(Zn2^2+^…N). If it is close to zero, both coordination bonds have the same lengths and thus contribute similarly to the polarization of the C-N and C=O bonds. In the NDM-1-ES, the distribution of the difference of coordination bond lengths is considerably shifted from zero. Thus, in the NDM-1, the effect of the Zn2^2+^...N coordination bond is more pronounced compared with the L1-ES. Indeed, the distance distribution of the C-N bond is wider and shifted to larger values; C-N bond lengths are observed for systems with shorter Zn2^2+^...N (Figure 4C).

For all considered interatomic distances, the same tendency is observed. In the L1-ES, distance distributions are narrower than for the NDM-1-ES. We suppose that this is due to the different organization of the active site region. In the L1, the substrate is additionally fixed by hydrophobic interactions with the Pro226 residue of the loop 10 (Figure 1). These interactions restrict significant movements of the imipenem in the active site of the L1. Conversely, in the NDM-1, Gly219 residue is located above the substrate (at the same position as Pro226 in the L1), and the loop 10 is located further from the substrate (Figure 1). Thus, in the NDM-1, the imipenem is generally more flexible compared with the L1 that is also pronounced in the distributions of key interatomic distances.

The detailed analysis of distance distributions for the NDM-1-ES (Figure 3) reveals that they are better described as a sum of normal distributions rather than one. Thus, structural flexibility of the active site leads to the heterogeneity of the ES complexes that is seen in the distributions of geometry parameters. Figure 4A and Table 1 demonstrate that there are three populations in the NDM-1 ES with respect to the C…O_w_ distance with similar weights. Similarly, multimodal distributions of other interatomic distances discussed above are observed. We found no good correlations between considered distances. The poor one was found between C-N bond lengths and the length of coordination bond that polarizes this bond (Figure 4C). Also, we found that, generally, structures with shorter distances of the nucleophilic attack have smaller coordination bond lengths (Figure 4B). It means that the active site is fluctuating between tighter states that are more reactive and looser conformations with less activated substrate. 

### 2.2. Dynamic Behavior of the ES Complexes: Electron Density Features

**Figure 5:**
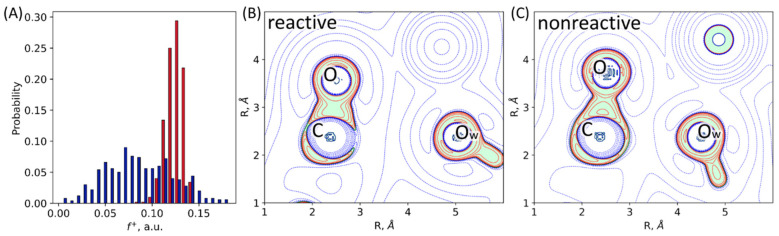
(**A**) Distributions of Fukui electrophilicity indices, $f^{+}$, on the carbonyl carbon atom of the imipenem in the ES complexes with the L1 (red) and NDM-1 (navy). (**B**,**C**) Representative Laplacian of the electron density maps in the plane of the carbonyl group of the substrate (C and O atoms) and an oxygen atom, O_w_, of the catalytic O_w_H^−^ discriminating, (**B**) reactive, and (**C**) nonreactive ES complexes. Contour lines for the Laplacian of electron density maps are ±(2; 4; 8)·10^n^ a.u., −2 ≤ n ≤ 1, blue dashed contour lines indicate the electron density depletion areas (∇^2^ρ(r) > 0), red solid lines identify the electron density concentration (∇^2^ρ(r) < 0), and green solid line corresponds to ∇^2^ρ(r) = 0. The area with ∇^2^ρ(r) < 0 is colored in light green.

Electron density descriptors, such as Fukui electrophilicity index and Laplacian of electron density, can quantify the activation of the substrate by the enzyme in its active site. We calculated Fukui electrophilicity indices at 500 MD frames for each system (Figure 5). Higher electrophilicity index of the carbonyl carbon atom of the imipenem is an indication of the more electrophilic atom, that is, such a carbon atom is more prepared for a nucleophilic attack. Fukui electrophilicity index distribution for the imipenem carbonyl carbon atom is narrower for the L1-ES (0.083–0.145 a.u.) than for the NDM-1-ES (0.002–0.181 a.u.), similarly to the distributions of geometry parameters discussed above (Figure 5A). Distributions are characterized by the following average values and standard deviations: 0.124 ± 0.009 a.u. for the L1 and 0.086 ± 0.036 a.u. for the NDM-1. The standard deviation of the Fukui electrophilicity index is much larger for the NDM-1, and the distribution is better described by three states. Indeed, we found three populations with the distributions centered at 0.047 a.u., 0.081 a.u., and 0.123 a.u., with the corresponding weights of 0.29, 0.38 and 0.33. The most reactive population of the NDM-1 has similar electrophilicity index distribution as the distribution of the L1 values.

Laplacian of electron density, ∇^2^ρ(r), maps in the plane of carbonyl group of the substrate (C and O atoms) and the nucleophilic atom, O_w_, allow binary classification of species as reactive and nonreactive. We calculated these 2D maps and classified each structure as either reactive or nonreactive for a set of 500 frames from QM/MM MD trajectory of the ES complexes of both enzymes. Reactive species (Figure 5B) are characterized by the electron density deconcentration (∇^2^ρ(r) > 0) in the carbonyl carbon atom region between the C and O_w_ nuclei. On the map, this is depicted as the area colored in white with blue dashed lines. For nonreactive species, the carbonyl carbon atom is enveloped by the electron density concentration region (∇^2^ρ(r) < 0, colored in light green with red solid lines, Figure 5C). We found that all considered frames from the QM/MM MD trajectory of the L1-ES complex are reactive (100%), and for the NDM-1-ES the major fraction is reactive (90%). 

Results of electron density analysis are interrelated with key interatomic distances in the active sites of two considered complexes. As noted in 3.1, the substrate is less flexible in the active site of the L1 compared with the NDM-1. For the L1-ES, narrow distributions of nucleophilic attack distances, coordination bonds, and bonds in the β-lactam ring are observed. In the NDM-1-ES, imipenem is more exposed to the solution and, generally, more flexible, that is, pronounced in considerably broader distributions of considered distances. The distributions of the cooperative action of coordination bonds in Figure 3F show that in the L1 both coordination bonds are strong, while the Zn2^2+^…N coordination bond dominates in the NDM-1. Hence, all these geometry features of the active site led to the different extent of substrate activation in the active site and different heterogeneity of states. In the L1-ES, 100% of states are reactive, while 90% are reactive in the NDM-1-ES. Fukui electrophilicity indices of the carbonyl carbon atoms are larger in the L1-ES compared with the NDM-1-ES. Also, for L1, we observe a unimodal distribution for both geometry and electron density features, whereas in the NDM-1 we find heterogeneity that can be described with at least 3 states.

### 2.3. Interrelation between Geometry and Electron Density Features in the ES

**Figure 6:**
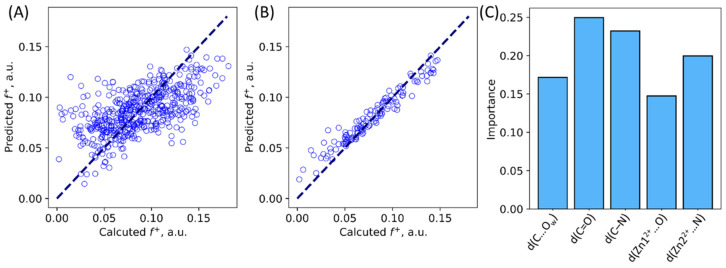
Calculated vs. predicted Fukui indices for the NDM-1—imipenem ES complexes. (**A**) Predictions made from multivariable linear regression. (**B**) Results for the validation set in the random forest machine learning algorithm. (**C**) Importance of geometry parameters suggested by machine learning.

Multiple variable linear regression analysis and machine learning random forest algorithm were utilized to study the relationship between geometry features and Fukui electrophilicity index of the carbonyl carbon atom in the ES state in the NDM-1. We selected the same geometry criteria as the ones shown in Figure 3. We obtained a multivariable linear regression with the MAE 0.022 a.u. (Figure 6A). This error is large, still, it allows to discriminate qualitatively both more and less reactive species. This is useful, as calculation of Fukui indices require additional computations in doublet state with an additional electron in the system. Still, herein, we are mostly interested in understanding the nature of the substrate activation, that is, which geometry parameters are more important. To do this, we utilized machine learning algorithms. Random forest approach resulted in a much better prediction quality of Fukui indices (Figure 6B), with the MAE 0.008 a.u. Also, it allowed us to explicitly estimate which geometry parameters are important (Figure 6C). We found that no geometry parameter has an importance exceeding 0.25; both distance of nucleophilic attack and polarization of the N-C=O fragment are important for the substrate activation. 

### 2.4. The First Reaction Step

**Figure 7:**
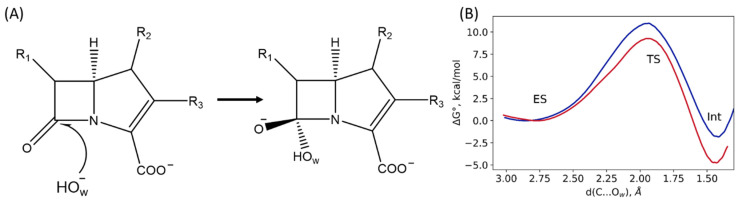
(**A**) Scheme of the first step of the reaction, black arrow indicates reaction coordinate of nucleophilic attack of the carbonyl carbon atom of the imipenem by the catalytic O_w_H^−^. (**B**) Gibbs energy profiles of the first step of the imipenem hydrolysis reaction in the active sites of the L1 (red, this study) and the NDM-1 (blue, ref. [20]).

The first step of the reaction is the nucleophilic attack of the hydroxide anion (O_w_H^−^) on the carbonyl carbon atom of the β-lactam ring (Figure 7A). Here, we perform a set of QM/MM MD simulations with harmonic potentials centered at different values of the reaction coordinate, d(C…O_w_). This allows us to obtain Gibbs energy profiles for the first step for both systems and compare them. We are interested in the comparison of the Gibbs energy profiles obtained for the NDM-1 with the L1-containing systems. The reaction coordinate at the first step in the umbrella sampling simulations is the distance of the nucleophilic attack, d(C…O_w_). Energy barriers at the first step are quite low, being 9 kcal/mol for the L1-containing system and 11 kcal/mol for the NDM-1. The difference between the energy barriers of the first step of the reaction in the L1 and the NDM-1 is about 2 kcal/mol, while the intermediate, Int, is 1.8 kcal/mol stabilized relative to the ES in the NDM-1 and almost 5 kcal/mol lower in energy in the L1 (Figure 7B). Thus, the first step of the reaction occurs more efficiently in the L1-imipenem complex. This is in line with the analysis of the ES complex: the carbonyl carbon atom of the substrate is more electrophilic due to the tighter and less flexible binding of the imipenem in the L1 active site. These results are in line with the data obtained on potential energy surface [8]. The energy barrier is lower, and the intermediate stabilization energy is larger in the L1 compared with the NDM-1.

## 3. Methods

The enzyme-substrate complex of the L1 and imipenem was obtained from the crystal structure PDB ID: 2AIO [26], and the enzyme-substrate complex of the NDM-1 and imipenem was obtained from the crystal structure PDB ID: 5YPK [27]. In the L1-containing system, hydrolyzed moxalactam was substituted with imipenem by superimposition of atoms of the hydrolyzed β-lactam ring and neighboring carboxylate group of moxalactam with the β-lactam ring and carboxylate group of imipenem. The complex with the NDM-1 imipenem was restored to its non-hydrolyzed form. Protonation states of acidic and basic amino acids were assigned to model neutral pH as follows. Carboxyl groups of aspartate and glutamate residues were negatively charged; side chains of lysine and arginine residues were in the protonated states; histidine residues were neutral and the protonation of either δ or ε nitrogen atom was chosen depending on the local environment. In the L1, His94, His118, and His 155 were N_ε_-protonated, others were N_δ_-protonated. In the NDM-1, all histidine residues, except for His122, were N_δ_-protonated. These systems were solvated in rectangular water boxes, so that the distance from the protein surface to the border of the cell exceeded 12 Å. These systems were neutralized by adding sodium ions. Systems were preliminary equilibrated during 10 ns in classical molecular dynamics (MD) simulations performed at T = 300 K and *p* = 1 atm with the 1 fs integration time step using CHARMM36 [28] force field for the protein, TIP3P [29] for water molecules, CGenFF [30] for the imipenem, and force filed parameters for zinc cations from Ref. [31]. Root-mean-square deviation (RMSD) values along the obtained trajectories showed that this length of the trajectories is enough for the complete relaxation of the system. Then the representative frames from the last 2 ns of the MD runs were used to prepare models for subsequent QM/MM MD simulations. 

The QM subsystem included the imipenem, two Zn^2+^ cations, and side chains of amino acid residues that form coordination bonds with them, catalytic hydroxide anion, and side chains that form hydrogen bonds with the imipenem (Ser221 and Tyr32 in the L1; Lys211, Leu218-Asn220, in the NDM-1). All QM/MM MD calculations were performed at the Kohm-Sham DFT level with the PBE0 functional [32] and imperial dispersion correction D3 [33], all electron 6-31G** basis set was utilized for all atom except for zinc, which was described with the pseudopotentials LANL2DZ [34]. QM/MM MD calculations were performed in the combination of TeraChem and NAMD programs [35]. The TeraChem program [36] was used to calculate forces in the QM region and NAMD in the MM part, as well as molecular dynamics steps [37]. QM/MM MD simulations were performed in the NPT ensemble at T = 300 K and *p* = 1 atm with the 1 fs integration time step. The cutoff distance for point charges of the MM subsystem contributing to the QM Hamiltonian was 12 Å. The VMD software [38] was used for preparation of the model systems and subsequent analysis of MD trajectories. Gibbs energy profiles for the first step, including nucleophilic attack and formation of the tetrahedral intermediate, were calculated using the umbrella sampling approach [39]. The sets of 5–10 ps runs were performed with harmonic potentials centered at different values of the reaction coordinate, which was the distance of nucleophilic attack. The force constant of the harmonic potential ½·K·(ξ − ξ_0_)^2^ was usually set to 40 kcal/mol/Å^2^, and additional trajectories with K = 80–320 kcal/mol/Å^2^ in transition state regions were calculated. Harmonic potentials were centered every 0.2 Å along the reaction coordinate. Umbrella integration (UI) and weighted histogram analysis method (WHAM) were utilized to reconstruct energy profiles of the first reaction steps. 

Analysis of dynamic behavior of the ES complexes was performed as follows. We selected sets of 500 frames for each model system from the last 5 ps QM/MM MD simulations of L1 and NMD-1 containing systems in the ES configuration. We estimated reactivity of imipenem using Laplacian of electron density maps calculated in the plane of carbonyl group of the substrate (C and O atoms) and the nucleophilic atom, O_w_, and Fukui electrophilicity index, *f*^+^, on the carbonyl carbon atom. Atomic index of electrophilicity, *f*^+^, is evaluated as a difference between Hirshfeld charges [40], calculated for the model system with N electrons (a system under configuration), and a system with the same geometry configuration, but with an additional electron. To do this, we performed additional single point calculations in the doublet state using unrestricted Kohn-Sham method with the same functional and basis set as calculations discussed above. Electron density analysis was performed in the Multiwfn program package [41].

Machine learning was performed for the datasets composed of calculated Fukui electrophilicity indices and key interatomic parameters at the corresponding MD frames. Random forest approach implemented in the Scikit-learn [42] was utilized to obtain a model to predict Fukui electrophilicity indices on the carbonyl carbon atom of imipenem from geometry parameters and to estimate importance of these parameters in value estimates. 

## 4. Conclusions

We performed molecular dynamic simulations with QM/MM potentials to compare structural flexibility of active site in the ES complex of two metallo-β-lactamases, the L1 and the NDM-1, with the imipenem antibiotic. Active sites in these complexes are covered by L10 loop that is more rigid in the L1 due to the presence of the proline residue. Analysis of key interatomic distances in the ES complex demonstrates a general trend: distributions of all distances are narrower in the L1-ES compared with the NDM-1-ES. Structural heterogeneity of ES complexes in the NDM-1 is also pronounced in the multimodal description of distance distributions. Distributions of the Fukui electrophilicity indices share the same tendencies: a narrow unimodal distribution in the L1, and a wide distribution composed of at least three populations with comparable weights in the NDM-1. Machine learning reveals that all geometry parameters, including distance of the nucleophilic attack, coordination bond lengths between the substrate and zinc cations, and C=O and C–N covalent bond lengths in the active site have similar importance for the Fukui index estimates. Gibbs energy profiles calculated for the hydrolysis of the imipenem in the active sites of the L1 and NDM-1 shares the same features with the profiles on the potential energy surface [8]. Formation of tighter and more rigid active site in the L1 leads to the lower energy barrier and larger extent of the intermediate stabilization relative to the ES complex.

## Figures and Tables

**Table 1:** Key interatomic distances obtained in the QM/MM MD simulations (with “MD” superscript) and at the minima on the potential energy surfaces corresponding to the ES complexes [8] (with “PES” superscript) for the L1-ES and the NDM-1-ES. In the rows with the QM/MM MD results the first line is for the range of corresponding distance and the second for the average value and standard deviation. The third line in the NDM-1^MD^ row corresponds to the decomposition of the distance distributions into three states (weight of each state is in parenthesis).

System	Distance, Å				
	d(C…O_w_)	d(Zn1^2+^…O)	d(Zn2^2+^…N)	d(C=O)	d(C-N)
**L1^MD^**	2.62–2.76	2.29–2.50	2.29–2.43	1.201–1.225	1.381–1.421
	2.68 ± 0.03	2.39 ± 0.04	2.36 ± 0.02	1.212 ± 0.003	1.402 ± 0.007
**L1^PES^**	2.84	2.43	2.75	1.214	1.388
**NDM-1^MD^**	2.57–3.30	2.65–3.94	2.23–3.13	1.150–1.270	1.324–1.564
	2.88 ± 0.15	3.23 ± 0.25	2.55 ± 0.18	1.210 ± 0.021	1.417 ± 0.036
	2.711 ± 0.004 (0.29) 2.876 ± 0.004 (0.42) 3.050 ± 0.007 (0.29)	2.99 ± 0.02 (0.30) 3.21 ± 0.02 (0.40) 3.49 ± 0.04 (0.30)	2.391 ± 0.005 (0.40) 2.579 ± 0.006 (0.39) 2.81 ± 0.02 (0.21)	1.1970 ± 0.0001 (0.28) 1.2094 ± 0.0001 (0.42) 1.2318 ± 0.0001 (0.30)	1.3847 ± 0.0007 (0.38) 1.4260 ± 0.0004 (0.43) 1.4628 ± 0.0009 (0.19)
**NDM-1^PES^**	2.74	3.59	2.24	1.200	1.455

## Data Availability

Not applicable.
